# The correlation between serum levels of alkaline phosphatase and bone mineral density in adults aged 20 to 59 years

**DOI:** 10.1097/MD.0000000000034755

**Published:** 2023-08-11

**Authors:** Xiaosong Cheng, Chengjin Zhao

**Affiliations:** a Medical School of Yan’an University, Yan’an, Shaanxi, China; b Affiliated Hospital of Yan’an University, Yan’an, Shaanxi, China.

**Keywords:** bone mineral density, NHANES, osteoporosis, serum alkaline phosphatase

## Abstract

Serum alkaline phosphatase (ALP) plays an important role in bone metabolism. However, the association between serum ALP and bone mineral density (BMD) remains inconclusive. Therefore, this study aimed to explore the relationship between serum ALP levels and pelvic BMD in young adults. We conducted a cross-sectional study using data from the National Health and Nutrition Examination Survey conducted from 2011 to 2016. Serum ALP levels and pelvic BMD were analyzed as independent and dependent variables, respectively. Weighted multivariate linear regression models and stratified analysis by age, sex, and race/ethnicity were applied after controlling for confounding factors to assess the relationship between serum ALP and pelvic BMD. Smooth curve fitting and threshold effect analysis were used to describe the nonlinear relationship between the 2 variables. A total of 7796 participants (4063 males and 3733 females) aged 20 to 59 years were included in this study. When serum ALP was represented as a continuous variable and fully adjusted in the regression model, ALP was significantly negatively correlated with pelvic BMD (β = −0.0008, 95% confidence interval: −0.0010 to −0.0006, *P* < .000001); this significant negative correlation persisted when ALP was transformed into a categorical variable, and was consistent in subgroup analyses. Additionally, smooth curve fitting and threshold effect analysis showed a persistent negative correlation between serum ALP and pelvic BMD, with a saturation effect at 97 U/L. Our results revealed a negative correlation between serum ALP levels and pelvic BMD in young adults. Monitoring serum ALP levels could help in the early detection of risks for bone metabolic disorders such as osteoporosis.

## 1. Introduction

Osteoporosis is a common metabolic bone disease caused by enhanced bone loss or inadequate bone formation, threatening individual health and quality of life.^[1,2]^ In the United States, osteoporosis leads to approximately 1.5 million fractures annually, predominantly in postmenopausal women.

The hallmark of this disease is low bone mass and deteriorated microstructure of bone tissue, resulting in reduced bone strength and increased risk of fragility fractures.^[3,4]^ bone mineral density (BMD) is an important indicator for assessing osteoporosis, as low BMD is associated with a high risk of fracture.^[5]^ The total cost of treatment and medication associated with osteoporosis increased by 64%, from 37.4 billion euros in 2010 to 56.9 billion euros in 2019, significantly burdening healthcare.^[6–8]^ Therefore, understanding the factors that affect BMD is of great clinical significance for the prevention and treatment of osteoporosis.

Alkaline phosphatase, a phosphomonoesterase present on the cell membrane, primarily catalyzes the hydrolysis of phosphate esters under alkaline conditions, releasing inorganic phosphate.^[9,10]^ It is highly expressed in tissues such as bones and liver. Furthermore, alkaline phosphatase (ALP) may be a key indicator of bone metabolism and BMD, as changes in its levels can reflect the growth, repair, and remodeling of bones.^[1]^ Therefore, this study aimed to investigate the correlation between ALP and pelvic BMD in adults, providing valuable insights for the prevention and treatment of osteoporosis.

## 2. Materials and methods

### 2.1. Study population

The National Health and Nutrition Examination Survey (NHANES) was a cross-sectional, stratified, multi-stage epidemiological study conducted in the United States, that assessed the health and nutritional status of adults and children. Written informed consent and assent were obtained from all adult participants and those under 18 years, respectively. NHANES has been approved by the Institutional Review Board of the National Center for Health Statistics, and all participants provided informed consent for their data to be utilized in subsequent research (https://www.cdc.gov/nchs/nhanes/irba98.htm). Our analysis uses data collected from 2011 to 2016, representing 3 dataset cycles of NHANES.

**Figure 1.**
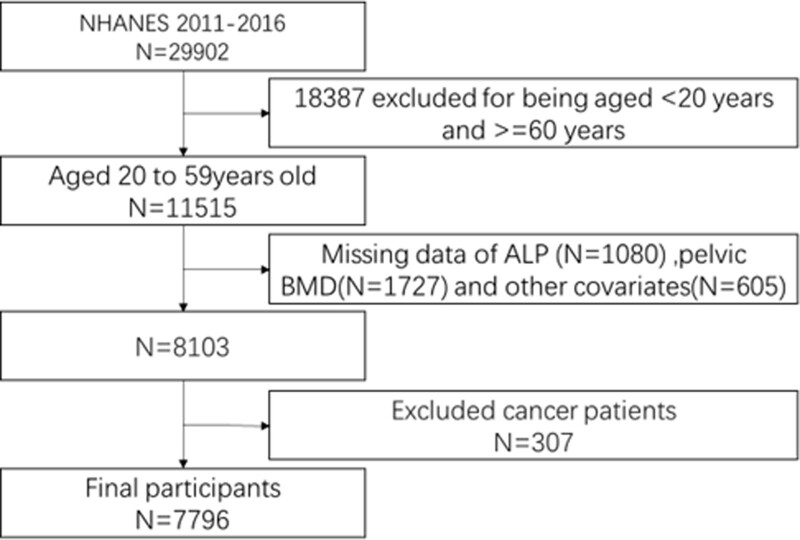
Flowchart of participant selection.

Of 29,902 participants, we excluded 18,387 individuals outside the 20 to 60-year age range, 1080 with missing data for serum levels of ALP, 1727 with missing pelvic BMD data, 307 with cancer, and 605 with missing data for relevant covariates. Finally, 7796 participants were included in this study (Fig. 1).

### 2.2. Study variables

The exposure variable in the study was serum ALP, analyzed using Beckman UniCel DxC 800 Synchron and Beckman UniCel DxC 660i Synchron Access Clinical Systems methods at Collaborative Laboratory Services, L.L. (https://wwwn.cdc.gov/Nchs/Nhanes/2015–2016/BIOPRO_I.htm). The studied outcome variable was pelvic BMD, measured by dual-energy x-ray absorptiometry scanning by trained and certified radiologic technologists using the Hologic Densitometer Discovery A (https://wwwn.cdc.gov/Nchs/Nhanes/2015–2016/DXX_I.htm).

The analyzed covariates included sex, age, race, education level, body mass index, smoking history (a smoker was defined as a person who smoked at least 100 cigarettes in life), alcohol consumption (a consumer was defined as a person who drank at least 12 alcoholic drinks in 1 year), and the levels of alanine aminotransferase, aspartate aminotransferase, lactate dehydrogenase, gamma-glutamyl transferase, triglycerides, uric acid, serum calcium, and serum phosphorus. Examination of the physiologic, clinical, and laboratory parameters was conducted by trained medical experts. More information on variable measurement is available on the NHANES website (https://www.cdc.gov/nchs/nhanes).

### 2.3. Data analysis

Data analysis was conducted using NHANES-weighted samples. Study participants were divided into quartiles based on their serum levels of ALP. Weighted multivariable linear regression models were used to analyze the relationship between serum ALP and pelvic BMD.

Weighted smoothed curve fitting and generalized additive models were used to analyze potential nonlinearity. If a nonlinear relationship was present, a 2-segment linear regression model was used to calculate threshold effects. Three models were established: Model I, unadjusted; Model II, minimally adjusted for sex, age, and race; Model III, fully adjusted. Data were analyzed using R (The R Foundation; http://www.r-project.org; version 3.4.3) and Empower (R) (www.empowerstats.net, X and Y Solutions, inc. Boston, Massachusetts). A *P* value < .05 (2-tailed) was considered statistically significant.

## 3. Results

### 3.1. Characteristics of the study population

This study included 7796 participants aged 20 to 59 years. The weighted characteristics of the study sample, including demographic and medical features, classified by serum ALP levels, are provided in Table 1 (Q1: 7–51 U/L; Q2: 52–62 U/L; Q3: 63–76 U/L; Q4: 77–352 U/L).

**Table 1:** Weighted characteristics of participants based on quartiles of serum alkaline phosphatase (U/L).

Serum ALP (U/L)	Total	Q1(7–51)	Q2(52–62)	Q3(63–76)	Q4(77–352)	*P* value
Age (yr, mean ± SD)	39.14 ± 11.65	37.69 ± 11.22	38.31 ± 11.28	39.49 ± 11.82	41.28 ± 12.00	<.0001
Sex (%)						<.0001
Male	52.79	46.58	55.43	56.33	53.01	
Female	47.21	53.42	44.57	43.67	46.99	
Race/ethnicity (%)						<.0001
Mexican American	10.45	5.59	8.69	11.30	16.97	
Non-Hispanic white	62.35	67.74	63.54	62.33	54.92	
Non-Hispanic black	11.69	11.67	11.63	10.92	12.67	
Other race/ethnicity	15.50	15.00	16.14	15.45	15.45	
Education level (%)						<.0001
Less than high school	13.62	8.76	12.64	14.77	18.95	
High school	20.92	16.50	19.31	23.03	25.33	
More than high school	65.45	74.74	68.05	62.20	55.72	
BMI (kg/m^2^, mean ± SD)	29.03 ± 6.82	27.21 ± 6.15	28.63 ± 6.55	29.55 ± 6.82	30.96 ± 7.23	<.0001
Smoking history (%)						<.0001
Yes	40.92	36.46	40.28	42.94	44.40	
No	59.08	63.54	59.72	57.06	55.60	
Alcohol consumption (%)						<.0001
Yes	81.02	83.03	82.47	81.63	76.45	
No	18.98	16.97	17.53	18.37	23.55	
ALT (U/L, mean ± SD)	26.65 ± 19.69	22.89 ± 15.56	25.33 ± 17.16	27.27 ± 17.95	31.70 ± 26.18	<.0001
AST (U/L, mean ± SD)	25.86 ± 17.71	23.40 ± 9.35	25.03 ± 12.34	25.76 ± 14.33	29.67 ± 29.14	<.0001
Lactate dehydrogenase (U/L, mean ± SD)	123.62 ± 25.37	117.95 ± 23.70	121.49 ± 23.84	125.37 ± 24.57	130.41 ± 27.79	<.0001
γ–glutamyl transferase (U/L, mean ± SD)	27.83 ± 43.62	20.06 ± 20.58	23.90 ± 29.52	27.97 ± 32.75	40.84 ± 73.53	<.0001
Triglycerides (mmol/L, mean ± SD)	1.74 ± 1.60	1.44 ± 1.68	1.65 ± 1.39	1.83 ± 1.59	2.07 ± 1.64	<.0001
Serum uric acid (umol/L, mean ± SD)	320.56 ± 80.44	306.14 ± 83.09	319.39 ± 79.56	326.22 ± 77.02	331.92 ± 79.59	<.0001
Serum calcium (mmol/L, mean ± SD)	2.35 ± 0.08	2.35 ± 0.08	2.35 ± 0.08	2.35 ± 0.08	2.35 ± 0.09	.1952
Serum phosphorus (mmol/L, mean ± SD)	1.21 ± 0.18	1.22 ± 0.18	1.22 ± 0.18	1.20 ± 0.18	1.21 ± 0.18	.0385
Pelvis BMD (g/cm^2^)	1.25 ± 0.16	1.26 ± 0.16	1.26 ± 0.16	1.25 ± 0.16	1.23 ± 0.16	<.0001

Mean ± SD for continuous variables: the *P* value was calculated by the weighted linear regression model. Percent (%) for categorical variables: the *P* value was calculated by the weighted chi-square test.

ALP = alkaline phosphatase, ALT = alanine aminotransferase, AST = aspartate aminotransferase, BMD = bone mineral density, BMI = body mass index.

The weighted mean age was 39.14 ± 11.65 years. Significant differences (*P* < .05) were observed in sex, age, race, education level, body mass index, smokers, alcohol consumers, and levels of alanine aminotransferase, aspartate aminotransferase, lactate dehydrogenase, gamma-glutamyl transferase, triglycerides, uric acid, and serum phosphorus, and pelvic BMD across different levels of serum ALP.

### 3.2. Relationship between serum levels of ALP and pelvic BMD

In Table 2, we reveal the negative correlation between serum ALP levels and pelvic BMD in Model 1 (−0.0005 [−0.0007,−0.0004] < 0.000001). This negative correlation persisted in the adjusted models [Model 2:−0.0005 (−0.0006,−0.0003) < 0.000001; Model 3:−0.0008 (−0.0010,−0.0006) < 0.000001]. In model 1, serum ALP levels in the Q2, Q3, and Q4 groups were all negatively correlated with pelvic BMD, although the correlation was not significant in the Q2 group. In the adjusted models, for all groups except for Q2 in Model 2 (−0.0074 [−0.0171, 0.0022] 0.131985), serum ALP levels were negatively correlated with pelvic BMD.

**Table 2:** The correlation between serum alkaline phosphatase (U/L) and pelvic bone mineral density (g/cm^2^).

	Model 1	Model 2	Model 3
	β (95% CI) *P* value	β (95% CI) *P* value	β (95% CI) *P* value
ALP (U/L)	−0.0005 (−0.0007, −0.0004) < .000001	−0.0005 (−0.0006, −0.0003) < .000001	−0.0008 (−0.0010, −0.0006) < .000001
Quintiles of ALP (U/L)			
Q1 (7–51)	Reference	Reference	Reference
Q2 (52–62)	−0.0032 (−0.0132,0.0069) .538228	−0.0074 (−0.0171,0.0022) .131985	−0.0133 (−0.0228, −0.0038) .006049
Q3 (63–76)	−0.0147 (−0.0246, −0.0048) .003517	−0.0175 (−0.0271, −0.0080) .000336	−0.0284 (−0.0379, −0.0189) < .000001
Q4 (77–352)	−0.0308 (−0.0410, −0.0205) < .000001	−0.0301 (−0.0401, −0.0201) < .000001	−0.0478 (−0.0580, −0.0376) < .000001
*P* for trend	<.001	<.001	<.001

Model 1: No covariates were adjusted. Model 2: Age, sex and race/ethnicity were adjusted. Model 3: Age, sex, race/ethnicity, education level, BMI, smoking history, alcohol consumption, ALT, AST, lactate dehydrogenase, γ-glutamyl transferase, triglycerides, serum uric acid, serum calcium and serum phosphorus were adjusted.

ALP = alkaline phosphatase, CI = confidence interval.

In Model 3, pelvic BMD decreased by 0.0478 g/cm^2^ in the Q4 group compared with Q1. In female adults, serum ALP levels were significantly negatively correlated with pelvic BMD. When stratified by age, pelvic BMD was higher in the 30 to 39 age group than in the 20 to 29 age group; however, it was significantly negatively correlated with serum ALP levels in the 50 to 59 age group.

The subgroup analysis (Table 3) revealed a persistent negative correlation between serum ALP levels and pelvic BMD across sex, age, and race-stratified subgroups.

**Table 3:** Stratified analysis of the correlation between serum alkaline phosphatase (U/L) and pelvic bone mineral density (g/cm^2^).

	Model 1	Model 2	Model 3
	β (95% CI) *P* value	β (95% CI) *P* value	β (95% CI) *P* value
Stratified by sex			
Male	−0.0005 (−0.0007, −0.0002) .0001	−0.0004 (−0.0007, −0.0002) .0004	−0.0006 (−0.0008, −0.0003) < .0001
Female	−0.0007 (−0.0009, −0.0004) < .0001	−0.0005 (−0.0007, −0.0002) < .0001	−0.0009 (−0.0012, −0.0007) < .0001
Stratified by age			
20–29 yr old	0.0000 (−0.0004, 0.0004) .8981	−0.0002 (−0.0006, 0.0002) .4191	−0.0007 (−0.0012, −0.0003) .0003
30–39 yr old	−0.0004 (−0.0007, −0.0001) .0087	−0.0004 (−0.0007, −0.0001) .0063	−0.0008 (−0.0011, −0.0005) < .0001
40–49 yr old	−0.0004 (−0.0007, −0.0001) 0.0105	−0.0005 (−0.0008, −0.0002) 0.0025	−0.0006 (−0.0009, −0.0002) 0.0007
50–59 yr old	−0.0007 (−0.0010, −0.0004) < .0001	−0.0004 (−0.0007, −0.0001) .0132	−0.0008 (−0.0011, −0.0004) < .0001
Stratified by race/ethnicity			
Mexican American	−0.0007 (−0.0011, −0.0004) .0002	−0.0007 (−0.0011, −0.0003) .0002	−0.0010 (−0.0014, −0.0005) < .0001
Non-Hispanic white	−0.0005 (−0.0007, −0.0002) .0007	−0.0004 (−0.0006, −0.0001) .0047	−0.0007 (−0.0010, −0.0004) < .0001
Non-Hispanic black	−0.0011 (−0.0015, −0.0006) < .0001	−0.0009 (−0.0013, −0.0005) < .0001	−0.0011 (−0.0015, −0.0007) < .0001
Other race/ethnicity	−0.0003 (−0.0007, 0.0000) .0516	−0.0004 (−0.0008, −0.0001) .0074	−0.0009 (−0.0013, −0.0006) < .0001

Model 1: No covariates were adjusted. Model 2: Age, sex and race/ethnicity were adjusted. Model 3: Age, sex, race/ethnicity, education level, BMI, smoking history, alcohol consumption, ALT, AST, lactate dehydrogenase, γ-glutamyl transferase, triglycerides, serum uric acid, serum calcium and serum phosphorus were adjusted.

CI = confidence interval.

### 3.3. Nonlinear relationship between serum levels of ALP and pelvic BMD

**Figure 2.**
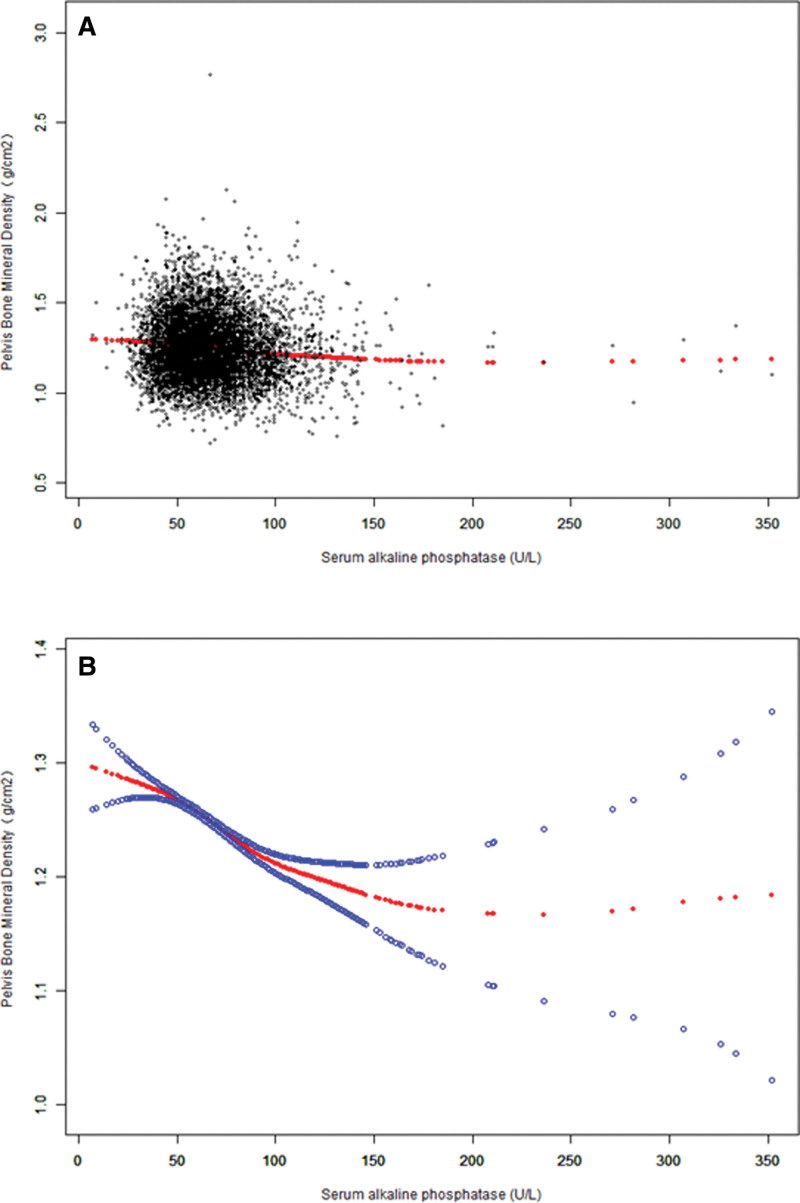
The correlation between serum ALP and pelvic BMD. (A) Each black point represents one participant serum ALP sample. (B) The solid red line illustrates the smooth fitting curve between variables, and two blue band illustrate the 95% confidence interval of the fitting. Age, sex, race/ethnicity, education level, BMI, smoking history, alcohol consumption, ALT, AST, lactate dehydrogenase, γ-glutamyl transferase, triglycerides, serum uric acid, serum calcium, and serum phosphorus were adjusted. ALP = alkaline phosphatase, ALT = alanine aminotransferase, AST = aspartate aminotransferase, BMD = bone mineral density, BMI = body mass index.

We further evaluated the nonlinear relationship between the 2 parameters using a smoothing curve fitting method, as shown in Figure 2.

**Figure 3.**
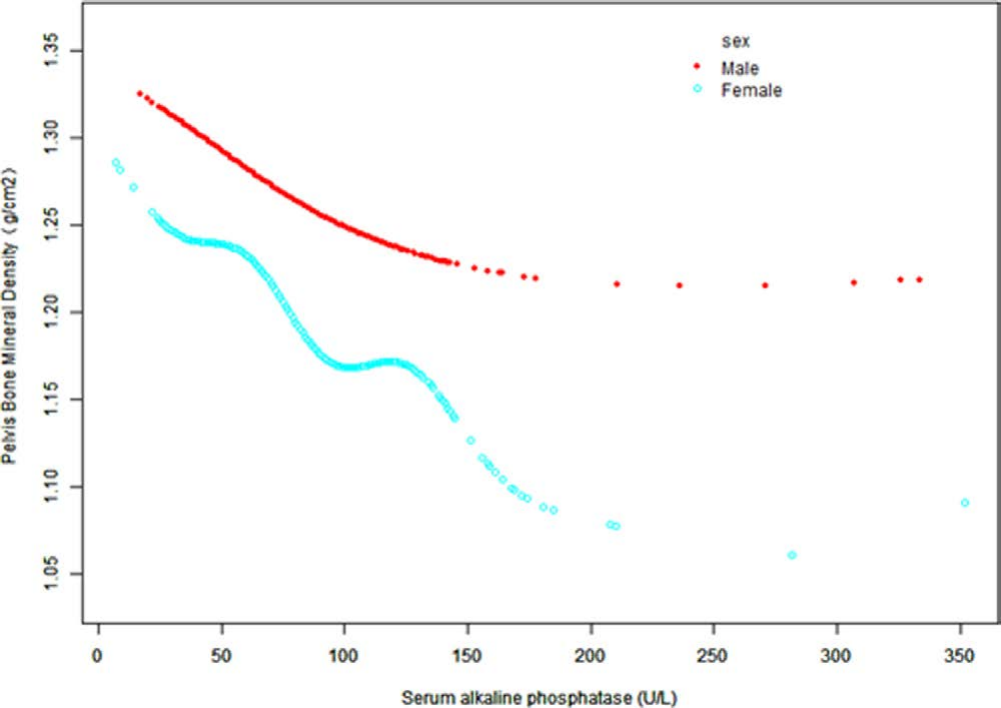
The correlation between serum ALP and pelvic BMD by sex. Age, race/ethnicity, education level, BMI, smoking history, alcohol consumption, ALT, AST, lactate dehydrogenase, γ-glutamyl transferase, triglycerides, serum uric acid, serum calcium, and serum phosphorus were adjusted. ALP = alkaline phosphatase, ALT = alanine aminotransferase, AST = aspartate aminotransferase, BMD = bone mineral density, BMI = body mass index.

**Figure 4.**
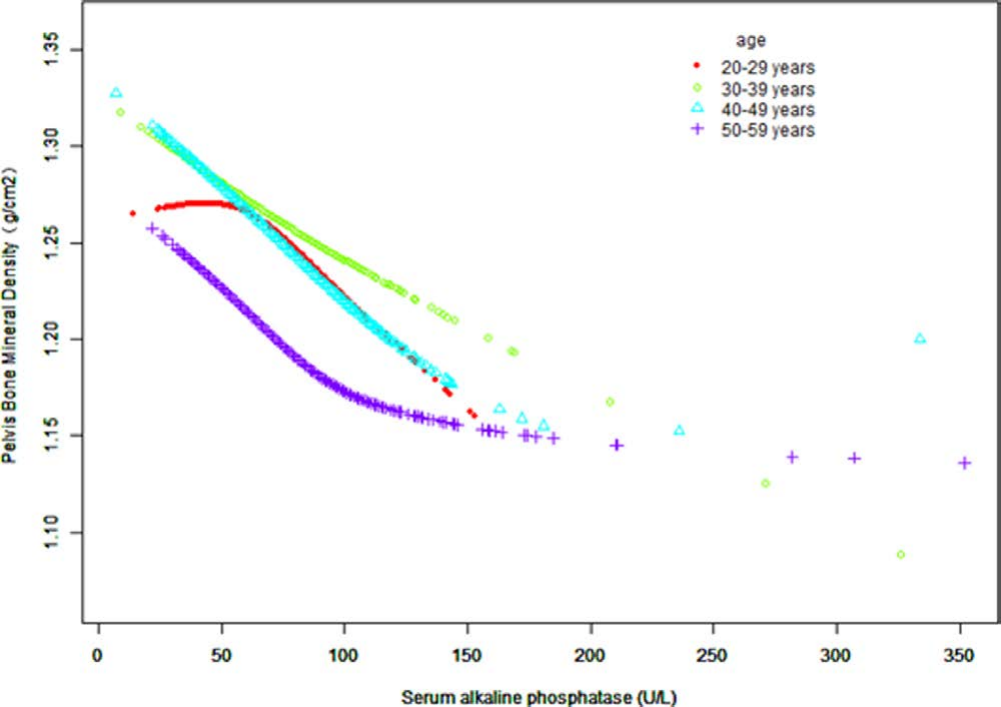
The correlation between serum ALP and pelvic BMD by age. Sex, race/ethnicity, education level, BMI, smoking history, alcohol consumption, ALT, AST, lactate dehydrogenase, γ-glutamyl transferase, triglycerides, serum uric acid, serum calcium, and serum phosphorus were adjusted. ALP = alkaline phosphatase, ALT = alanine aminotransferase, AST = aspartate aminotransferase, BMD = bone mineral density, BMI = body mass index.

**Figure 5.**
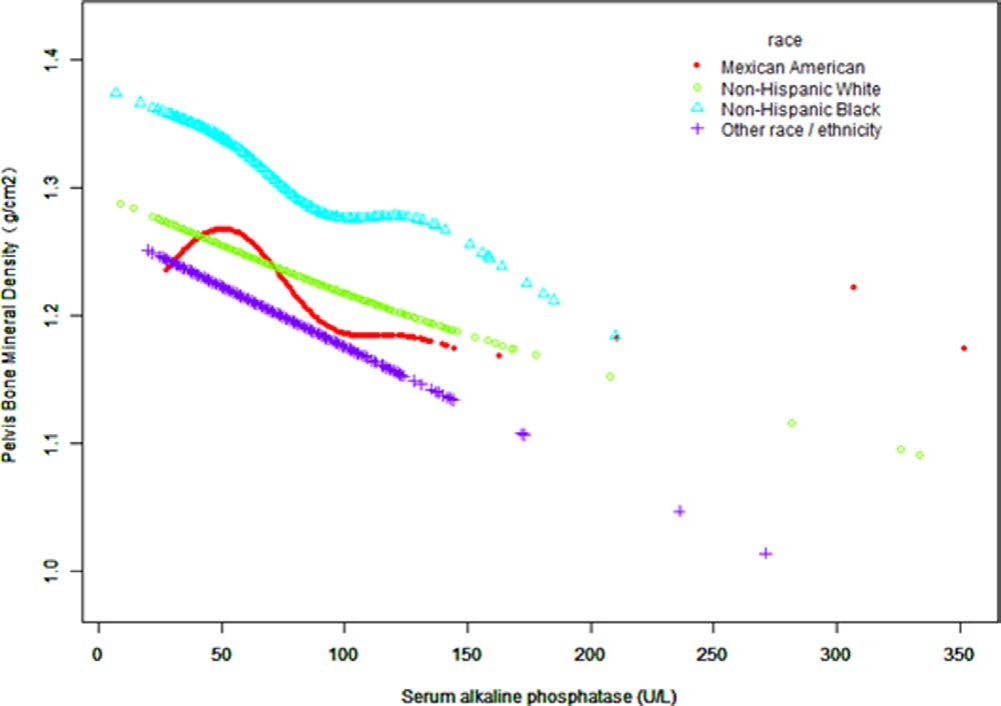
The correlation between serum ALP and pelvic BMD by race. Age, sex, education level, BMI, smoking history, alcohol consumption, ALT, AST, lactate dehydrogenase, γ-glutamyl transferase, triglycerides, serum uric acid, serum calcium, and serum phosphorus were adjusted. ALP = alkaline phosphatase, ALT = alanine aminotransferase, AST = aspartate aminotransferase, BMD = bone mineral density, BMI = body mass index.

Figure 3–5 display fitted smoothing curves and generalized additive models describing the linear or nonlinear relationships between serum ALP and pelvic BMD. A 2-segment linear regression model revealed a saturation effect value of 97 U/L between them, with an effect value of −0.0010 for serum ALP levels below 97 U/L and −0.0002 for serum ALP levels higher than 97 U/L, as shown in Table 4.

**Table 4:** Threshold effect analysis of serum alkaline phosphatase (U/L) and pelvic bone mineral density (g/cm^2^) using the two-piecewise linear regression model.

Pelvic BMD	Adjusted β (95% CI) *P* value
Fitting by the standard linear model	0.0008 (0.0003,0.0013) .0008
Fitting by the two-piecewise linear model	
Inflection point	97
Serum ALP < 97 U/L	−0.0010 (−0.0012, −0.0008) < .0001
Serum ALP > 97 U/L	−0.0002 (−0.0006,0.0002) .2525
Log likelihood ratio	<.001

Age, sex, race/ethnicity, education level, BMI, smoking history, alcohol consumption, ALT, AST, lactate dehydrogenase, γ-glutamyl transferase, triglycerides, serum uric acid, serum calcium and serum phosphorus were adjusted.

ALP = alkaline phosphatase, BMD = bone mineral density, CI = confidence interval.

## 4. Discussion

ALP is an enzyme widely present in tissues such as the skeletal system, liver, intestines, and placenta, and in the blood.^[11,12]^ Recently, its serum levels were correlated with bone metabolism and BMD.^[13–17]^ Our study investigated this correlation in adults aged 20 to 59 years and found a negative correlation between serum ALP levels and pelvic BMD, which persisted after adjusting for confounders (Table 2).

Females exhibited a more significant negative correlation between serum ALP levels and pelvic BMD than that exhibited by males. These results are consistent with those of a study from Japan that associates increased ALP levels in females with more severe osteoporosis.^[16]^ Age stratification indicated that pelvic BMD increased in the 30 to 39 age group compared with the 20 to 29 age group, while a significant negative correlation was observed in the 50 to 59 age group.

Osteoporosis is characterized by enhanced loss of bone mass or insufficient bone formation, which are reflected by BMD. ALP mainly hydrolyzes phosphate esters during bone formation, providing the necessary phosphate for hydroxyapatite deposition; it also hydrolyzes pyrophosphate, relieving its inhibitory effect on bone salt formation, and promoting bone formation.^[18,19]^ Thus, it can serve as a bone turnover marker.

Normal bone metabolism is maintained by a balance between osteoclastic bone resorption and osteoblastic bone formation, ensuring stable bone mass. When BMD is low, quiescent osteoblasts activate, resulting in unmineralized bone-like tissue and undifferentiated osteoblasts. These osteoblasts proliferate in a feedback manner and synthesize a large amount of bone alkaline phosphatase (BALP), significantly increasing serum ALP levels.^[20]^

Our research, based on a nationally representative sample, is highly relevant to the U.S. population. Additionally, the large sample size enabled subgroup analysis, reporting correlations between serum levels of ALP and pelvic BMD across sex, age, and racial/ethnic groups. However, the specific mechanisms underlying this relationship warrant further investigation. This study only included adults aged 20 to 59 years, and the correlation between ALP and BMD may vary in other age groups. Furthermore, this study used serum ALP, not BALP (an alkaline phosphatase isoenzyme with higher specificity in reflecting bone cell activity and bone formation). BALP secretion by bone tissue increases when calcium salt bone deposition is insufficient and decreases when the bone concentration of calcium salt is sufficient.^[9,10,21]^ Therefore, future research should also focus on the relationship between BALP and BMD.

Although current research lacks consistent conclusions, ALP remains a valuable clinical biomarker. In clinical practice, monitoring serum ALP levels can help physicians to evaluate bone metabolism status and identify early-stage bone metabolic disorders such as osteoporosis.

## 5. Conclusion

Our research indicates a negative correlation between serum ALP levels and pelvic BMD in the US adult population aged 20 to 59 years. Elevated serum ALP levels may indicate an increased risk of osteoporosis or decreased bone density and could potentially serve as a biomarker for the diagnosis and treatment of osteoporosis. However, further basic and clinical research is needed to elucidate the exact impact of ALP on BMD and to understand its underlying mechanisms.

## Acknowledgements

We express our gratitude to all NHANES staff for their dedication and efforts.

## Author contributions

**Data curation:** Xiaosong Cheng.

**Writing – original draft:** Xiaosong Cheng.

**Writing – review & editing:** Chengjin Zhao.
